# Metabolism in Retinopathy of Prematurity

**DOI:** 10.3390/life11111119

**Published:** 2021-10-21

**Authors:** Yohei Tomita, Ayumi Usui-Ouchi, Anders K. Nilsson, Jay Yang, Minji Ko, Ann Hellström, Zhongjie Fu

**Affiliations:** 1 Department of Ophthalmology, Boston Children’s Hospital, Harvard Medical School, Boston, MA 02115, USA; yohei.tomita@childrens.harvard.edu (Y.T.); jay.yang@childrens.harvard.edu (J.Y.); minji.ko@childrens.harvard.edu (M.K.); 2 Department of Ophthalmology, Juntendo University Urayasu Hospital, Chiba 279-0021, Japan; ausui@juntendo.ac.jp; 3 Department of Clinical Neuroscience, Institute of Neuroscience and Physiology, Sahlgrenska Academy, University of Gothenburg, 413 19 Gothenburg, Sweden; anders.k.nilsson@gu.se (A.K.N.); ann.hellstrom@medfak.gu.se (A.H.)

**Keywords:** retinopathy of prematurity, neovascularization, retinal metabolism, hyperglycemia, dyslipidemia, oxygen-induced retinopathy, hyperglycemia-associated retinopathy

## Abstract

Retinopathy of prematurity is defined as retinal abnormalities that occur during development as a consequence of disturbed oxygen conditions and nutrient supply after preterm birth. Both neuronal maturation and retinal vascularization are impaired, leading to the compensatory but uncontrolled retinal neovessel growth. Current therapeutic interventions target the hypoxia-induced neovessels but negatively impact retinal neurons and normal vessels. Emerging evidence suggests that metabolic disturbance is a significant and underexplored risk factor in the disease pathogenesis. Hyperglycemia and dyslipidemia correlate with the retinal neurovascular dysfunction in infants born prematurely. Nutritional and hormonal supplementation relieve metabolic stress and improve retinal maturation. Here we focus on the mechanisms through which metabolism is involved in preterm-birth-related retinal disorder from clinical and experimental investigations. We will review and discuss potential therapeutic targets through the restoration of metabolic responses to prevent disease development and progression.

## 1. Introduction

**Figure 1:**
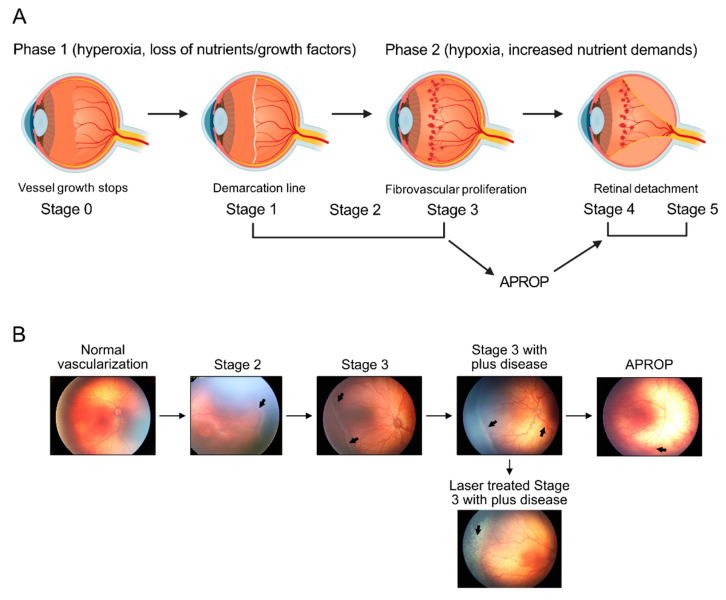
ROP progression in premature infants. (**A**) Schematics of the progression of human retinopathy of prematurity (ROP). Phases 1 and 2 of ROP are associated with different oxygen levels. Loss of essential nutrients and pro-angiogenic growth factors after birth in combination with provision of high supplemental oxygen, leads to hyperoxia that suppresses retinal vascularization (Phase 1). In the second phase of ROP (Phase 2), relative hypoxia and increased nutrient demands of the avascular retina drives fibrovascular proliferation. ROP Phase 2 is defined by anatomic changes, such as the demarcation line (stage 1), ridge (stage 2), extraretinal fibrovascular proliferation (stage 3), partial retinal detachment (stage 4), and total retinal detachment (stage 5). Any stage can develop into aggressive posterior ROP (APROP), which rapidly progresses to tractional retinal detachment (stage 4 or 5). Image made with graphics from ©BioRender (https://biorender.com/ (accessed on 18 October 2021) Agreement number: IA22XF3W0H) (**B**) Illustration of retinopathy of prematurity (ROP) development, from normal retinal neuro-vascular development, via stage 2 with ridge (arrow), stage 3 with neovascularization and hemorrhage (arrows), stage 3 with plus disease (arrow), APROP with central changes (arrow) and laser treatment (arrow) of stage 3 ROP.

Retinopathy of prematurity (ROP) is a leading cause of blindness in children worldwide, [1] and about 14,000–16,000 infants develop ROP in the US every year. After preterm birth, ROP begins with suppression in the growth of immature retinal vasculature (phase I ROP) (Figure 1A,B), secondary to oxygen supplementation and loss of growth factors normally provided in utero [2]. As the neural retina slowly matures, the increased metabolic demand for nutrients and oxygen is not met in the avascular retinal region. Hypoxia and nutrient deprivation are driving forces to induce retinal vessel growth [3,4]. However, these newly-formed vessels are uncontrolled and fragile (phase II ROP). Phase II ROP starts at postmenstrual age 30–32 weeks, which coincides with the rapid development of rod photoreceptors [5,6]. In a rat model of ROP, early photoreceptor dysfunction also predicts subsequent neovascularization [7]. Therefore, modulating retinal metabolic needs may preserve neuronal function and prevent pathologic angiogenesis. Emerging investigations of ROP metabolic changes have been reported with a focus on nutritional interventions such as essential omega-3 and omega-6 long-chain polyunsaturated fatty acids (LCPUFA), insulin-like growth factor 1 (IGF-1), and adiponectin [8,9,10]. Recently, novel blood metabolic biomarkers for ROP have been identified with metabolomics and lipidomics to predict ROP incidence and severity. In this review, we will summarize our current knowledge of metabolic changes and modulations in ROP gained from clinical and experimental investigations.

## 2. Clinical Investigations of Metabolic Changes in ROP

Poor postnatal weight gain predicts severe ROP in preterm infants [11,12]. Thus, improving nutritional support may improve weight gain and subsequently prevent ROP. The LACTACOL trial investigated growth rate in a cohort of preterm infants (gestational age [GA] 30–31 weeks) who were fed their own mother’s breast milk throughout the hospital stay (49–51 days) in relation to the metabolic signature of the maternal milk. The milk from the mothers of faster-growing infants contained more arginine, tyrosine, medium-chain saturated fatty acid, and omega-3 LCPUFA (docosahexaenoic acid (DHA), eicosapentaenoic acid (EPA)), as well as less glycine, taurine, and oleic/cis-vaccenic acid [13,14]. LCPUFA (omega-3, omega-6, and omega-9) in the milk from mothers who deliver before 28 weeks of pregnancy declines rapidly between postnatal day 7 and a postmenstrual age of 40 weeks, suggesting that this already low source of LCPUFA becomes increasingly inadequate to support the development of the preterm infant [15]. LCPUFA shortage is associated with ROP progression [8,9]. Specifically, both omega-3 and omega-6 LCPUFA and their relative distribution are likely necessary factors that promote normal vessel formation and prevent ROP [16]. A low intake of lipids, carbohydrates, and total calories correlates with an increased risk of severe ROP in preterm infants with GA <28 weeks in the ELGAN study [17]. Further elucidation of the nutrients and metabolites associated with ROP would help optimize maternal diet and parenteral nutrition, as well as personalize the nutritional care of preterm infants to prevent ROP. However, there are very limited studies that correlate blood metabolites with ROP in premature infants at the current stage.

### 2.1. Lipidomics

Postnatal blood levels of essential fatty acids DHA and arachidonic acid (AA) are low in premature infants and are correlated with ROP progression [8,9,18]. Clinical trials supplementing preterm infants with DHA to improve the development of visual function and prevent ROP have yielded inconsistent results. Two strategies have been used to restore infant DHA: (I) administration via intravenous lipid emulsions containing fish oil [19,20,21,22] or (II) enteral supplementation using DHA from single-cell oils or fish oil [23,24,25]. Most studies assessing the effect of DHA-rich intravenous lipid emulsions have been retrospective comparative studies while only a few have prospectively investigated their role in ROP outcome, which likely contributes to the heterogeneity of the reported results. Another limitation of intravenous supplementation is duration, as preterm infants only rely on parenteral nutrition for a limited time, usually in the range of a few days to weeks. A recent Cochrane review found no support that the use of fish oil containing lipid emulsions compared to non-fish oil lipid emulsions in preterm infants reduces severe ROP (stage 3 or greater or requiring surgery), although the evidence was very low quality [26]. Contrary, a meta-analysis comparing pooled results from randomized clinical trials of early administration of fish oil vs. non-fish oil lipid emulsions found a significant reduction in the relative risk of severe ROP favoring fish oil lipids [27]. A potentially negative aspect of supplementing preterm infants with fish oil, which is naturally rich in omega-3 EPA and DHA but relatively low in omega-6 AA, is that it causes a decrease in circulating AA [24,28,29]. Daily enteral DHA and AA (100 mg and 50 mg/kg/day, respectively) given to preterm infants from birth to term equivalent age increases circulating DHA and AA levels and reduces severe ROP [25,30]. Enteral DHA supplementation at 75 mg/kg/day to preterm infants for two weeks significantly lowers the risk for stage 3 ROP [23]. Full-term infants supplemented with DHA:AA in the formula at a 1:2 ratio improves visual acuity [31]. However, doubling or tripling DHA does not confer additional benefits [31]. Meta-analysis of the randomized comparisons of DHA-supplemented formula vs. DHA-free formula to preterm infants shows improved visual resolution acuity at 2 and 4 months of corrected age [32]. In other studies, very preterm infants supplemented with DHA in the first months of life do not have better visual processing [22,33].

In mouse ROP, dietary DHA has been found to decrease retinal neovascularization [9,34,35]. DHA metabolites via lipoxygenase (LOX) inhibit while AA metabolites via LOX induce retinal angiogenesis [36]. However, both DHA and AA metabolites via cytochrome P450 oxidases (CYP) exert pro-angiogenic effects in increasing retinal neovascularization [37,38]. Interestingly, the DHA-derived diol 19,20-dihydroxydocosapentaenoic acid (19,20-DHDP) via the soluble epoxide hydrolase (sEH) pathway reduces retinal neovascularization and prevents astrocytic loss by targeting the mitochondrial membrane [39]. Moreover, both dietary DHA (no AA) and AA (no DHA) in rats profoundly alter cardiac mitochondrial phospholipid fatty acid compositions and suppress Ca^2+^-induced opening of the mitochondrial permeability transition pore with cell death [40,41]. Dietary DHA (no AA) also depletes cardiac mitochondrial AA content [40]. These findings suggest that the impacts of dietary DHA on ROP might be influenced by the balance between DHA and AA, as well as DHA and AA metabolites via LOX and CYP pathways.

**Figure 2:**
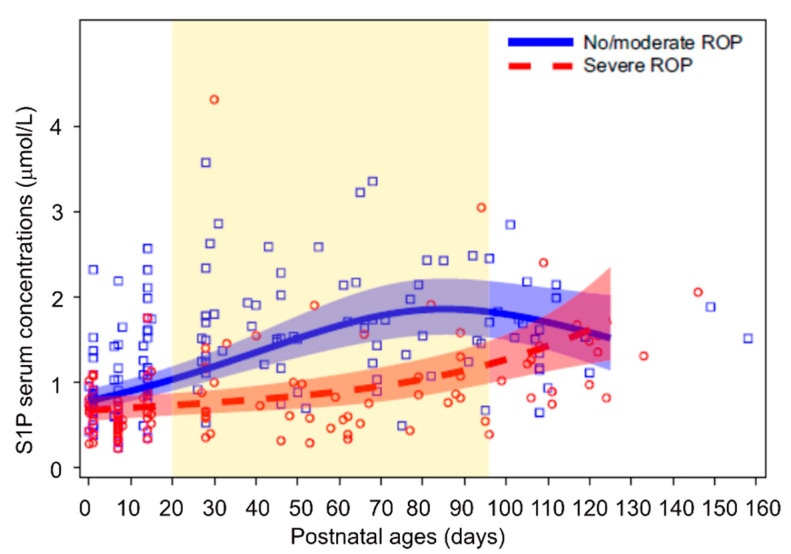
Serum S1P levels and ROP in premature infants. Dots show measured serum S1P levels and lines (with 95% CI) represent estimates from mixed model for repeated measures adjusted for GA at birth and weight standard deviation score. Graph area highlighted in yellow represents time points where curves differ significantly after adjustment for multiplicity. *n* = 28 for no/moderate ROP, *n* = 19 for severe ROP. Graph was adapted from [42].

Nilsson et al. showed the correlation of serum sphingolipids with ROP in 47 preterm infants born at GA < 28 weeks [42]. Low postnatal sphingosine-1-phosphate (S1P) levels are strongly associated with severe ROP after adjusting for GA and birth weight (Figure 2) [42]. S1P is a lysophospholipid and serves as a bioactive lipid mediator for intracellular and extracellular signals [43,44]. S1P signaling is needed for retinal vascular specialization, and the loss of S1P receptors causes extremely dense and disorganized retinal vascular plexi during development [45]. However, blockade of S1P with sonepcizumab suppresses hypoxia-induced retinal neovascularization in mouse ROP [46]. Further investigations are needed to explore the role of S1P signaling in ROP severity.

### 2.2. Proteomics

Lynch et al. reported that low levels of plasma mitochondrial superoxide dismutase (MnSOD) within the first postnatal week are associated with increased risk of severe ROP in 35 preterm infants with GA < 29 weeks [47], suggesting potentially disturbed metabolic status in ROP infants. SOD is a strong antioxidant in scavenging oxygen radicals generated from metabolic processes, and high SOD activity ameliorates pathological retinal angiogenesis in mice modeling phase II ROP [48]. Increased peroxidant antioxidant balance predicts the severity of ROP [49]. Prooxidant parameters including total oxidative status and malondialdehyde are higher in infants with ROP vs no ROP [50]. Furthermore, preterm infants with ROP have lower levels of the antioxidant glutathione (GSH) in their red blood cells during the first two weeks of life [51]. Premature infants are susceptible to oxygen-related damage due to their low levels of antioxidants (vitamin A and E, SOD, and catalase) [52]. Results from a recent pilot study in preterm infants suggests that enteral supplementation with AA and DHA in a 2:1 ratio can improve the antioxidant to oxidant balance [53]. Danielsson et al. further profiled the longitudinal serum protein levels between postnatal day 1 and postmenstrual age 40 weeks in 14 preterm infants with GA 22.9 to 27.6 weeks [54]. Serum proteins, such as AGER, ANGPT1, APP, CD40LG, GDF2, HBEGF, MMP12, and SERPINE1 involved in lipid metabolism are persistently lower in patients who develop severe ROP [54], suggesting a disturbed lipid metabolic status in ROP.

### 2.3. Metabolomics

Yang et al. reported that blood malonylcarnitine (C3DC) and glycine are higher in ROP (40 infants, 15 males, and 25 females) vs. non-ROP controls (41 infants, 30 males, 11 females) after adjusting for sex [55]. C3DC is produced from malonyl-coenzyme A (CoA), and C3DC levels reflect malonyl-CoA as patients born with malonyl-CoA decarboxylase deficiency have elevated C3DC [56,57]. Therefore, high blood C3DC levels in ROP infants indicate high concentrations of malonyl-CoA and potential disruptions of fatty acid oxidation, as malonyl-CoA inhibits carnitine palmitoyltransferase 1A (CPT1A, transporting lipids into mitochondria) [58]. Pathological angiogenesis is induced in mouse retinas with low lipid uptake and reduced fatty acid oxidation [3]. Therefore, restoration of fatty acid oxidation may prevent ROP progression.

Meanwhile, Zhou et al. also found that 11 out of 29 significantly altered blood metabolites between severe ROP (38 cases) vs. age-matched infants (23 cases) are amino acids and their derivatives [59]. Elevated plasma amino acids such as citrulline, proline, threonine, and tryptophan in ROP patients are also observed in retinas from mice modeling phase II ROP [59,60,61]. Experimental evidence shows a significant contribution of amino acids (such as proline, arginine, and glutamine) to retinal vascular function [62,63,64]. Further exploration of amino acid metabolism in ROP may identify new biomarkers for the disease development and progression, as well as uncover new therapeutic targets.

Together, clinical investigations suggest a prominent role of lipid and amino acid metabolism in ROP. Some but not all have experimental evidence. Further validation with increased clinical cases and experimental examination is needed to confirm these findings.

## 3. Experimental Investigations of Retinal Metabolism in ROP

### 3.1. Oxygen-Induced Retinopathy (OIR)

**Figure 3:**
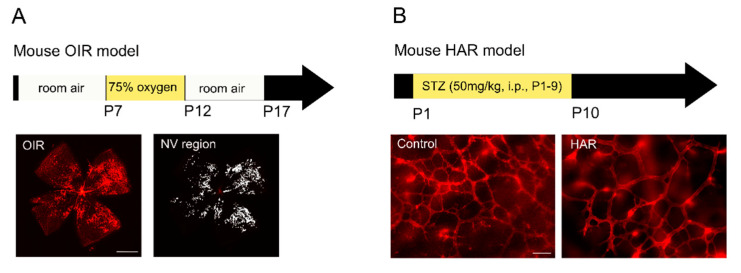
Mouse models of ROP. (**A**) Mouse model of oxygen-induced retinopathy (OIR). Mouse neonates at postnatal day (P) 7 with their nursing dam are exposed to 75% oxygen for five days. At P12, mice are returned to room air. At P17, neovascularization (NV) reaches the maximum. Retinal vasculature is visualized with isolectin (red) staining and NV area is highlighted in white. Scale bar, 1 mm. (**B**) Mouse model of hyperglycemia-associated retinopathy (HAR). Hyperglycemia is induced in mouse neonates with streptozotocin (STZ, 50 mg/kg) intraperitoneally (i.p.) from P1 to P9. At P10, retinal vessels in the deep vascular plexus are visualized with isolectin (red) staining. Reduced retinal vascular density is observed in HAR. Scale bar, 50 µm.

OIR has been developed in various species, such as in dogs [65], cats [66], rats [67] and mice [4] to mimic human ROP. The mouse OIR model (Figure 3A), with the advantage of genetic manipulation, has been widely used to study retinal vascular and neuronal changes in ROP. Mouse neonates with their nursing dam at postnatal day (P) 7 are exposed to 75% oxygen for five days and returned to room air (21% oxygen) at P12 [4]. Hyperoxic exposure induced retinal vessel loss and the relative hypoxia-induced retinal neovascularization reaches maximal levels at P17 [4]. This model has contributed to the developing of anti-vascular endothelial growth factor (anti-VEGFA) therapy to improve retinal neovascular diseases [68,69,70,71]. Hypoxia-inducible factor (HIF), a transcriptional factor responding to hypoxia in the tissue, regulates angiogenic genes such as VEGFA [72,73,74]. Miwa et al. reported that topotecan (HIF inhibitor) administered during the hypoxic phase (P12 to P16) suppresses the HIF pathway and the expression of *Vegf*, resulting in the prevention of retinal neovascularization in OIR mice [75]. Usui-Ouchi et al. reported that intravitreal injection of peptides derived from intrinsically disordered protein CITED2, a negative feedback regulator for HIF activation, inhibited retinal neovascularization and vaso-obliteration in OIR [76]. Meanwhile, Hoppe et al. suggested that stabilizing HIF-1 during the hyperoxic phase prevents vaso-obliteration and subsequent neovascularization in OIR mice [77]. Elevated aerobic glycolysis in response to HIF stabilization with HIF prolyl hydroxylase inhibitors before or during hyperoxia might contribute to the neurovascular protection of retina in OIR mice [78,79]. In addition, serine metabolism is also required for HIF-1 mediated protection against retinopathy in OIR mice [78]. These studies demonstrate HIF as a crucial factor for retinal angiogenesis and its metabolic modulation of the retina in early ROP. Inducing aerobic glycolysis and modulation of serine metabolism may prevent hyperoxia-induced retinal vessel loss in ROP.

Metabolomics profiling of the retina in OIR mice reveals disrupted glycine/creatine pathway with high retinal glycine and low creatine levels [60]. Supplementation of creatine during the hypoxic phase (P12 to P16) inhibits retinal neovascularization in OIR mice [60]. Recent studies also suggest that low glycine and serine levels are correlated with retinal degeneration [80,81]. Glycine promotes angiogenesis in mouse hind-limb ischemia in vivo and in human umbilical vein endothelial cells in vitro, as well as protects endothelial cell mitochondrial function [82]. Together, the glycine-creatine pathway may have a crucial role in ROP development.

Paris et al. reported that increases in arginine-to-proline pathway and other metabolites in the urea cycle, and decrease in purine metabolism in the whole eye from OIR versus control mice [61]. Lu et al. also showed induced plasma proline, ornithine, and glutamine, which are essential components of the arginine and proline pathway in OIR rats [83]. Systemic administration of the dipeptide arginyl-glutamine during hypoxia (P12 to P17) inhibits retinal neovascularization by ~80% and decreases neovascular tuft leakage in OIR mice [63]. Taken together, these results may suggest arginine and proline pathways as the potential diagnosis and treatments for ROP.

### 3.2. Hyperglycemia-Associated Retinopathy (HAR)

In addition to oxygen, hyperglycemia, which commonly occurs in preterm infants (~80% with birth weight <750 g and ~45% with birth weight <1000 g) [84], is the other significant risk factor for ROP. Hyperglycemia, particularly in the first postnatal weeks, highly correlates with delayed retinal vascularization [85] and ROP progression in preterm infants [86,87,88,89,90,91,92,93,94,95,96]. However, hyperglycemia is understudied as the current OIR model has limitations in mimicking the hyperglycemic aspect of ROP. In patients with diabetes, about one-third develop some signs of diabetic retinopathy [97], the leading cause of blindness in working-aged people [98]. Therefore, there is an urgent need to better understand the impacts of postnatal hyperglycemia on ROP. The mouse model of HAR (Figure 3B) [99] is established to investigate the impacts of metabolic dysregulation on retinal vessels and neurons at the early stages of development.

In mouse HAR model [99], hyperglycemia is induced with daily intraperitoneal injection of streptozotocin (50 mg/kg) from P1 to P9. High blood glucose is observed around P8. At P10, deep retinal vascular plexus formation between the inner nuclear layer and the photoreceptors is delayed along with the induction of hyperglycemia. Insulin treatment from P7 to P9 partially reverses the delay in retinal vessel growth. At P30, there is remarkable decrease in retinal neuronal function and retinal thickness. These observations suggest that hyperglycemia in early postnatal days induces retinal vascular and neuronal pathology, corresponding to the strong correlation between postnatal hyperglycemia and ROP progression in preterm infants. The mouse HAR model is a feasible tool to explore clinical risk factors for early ROP and potential therapeutic interventions to prevent disease progression. In preterm infants, hyperglycemia positively correlates with low serum adiponectin (APN) levels, and low serum APN levels positively correlates with delayed retinal vascularization (phase 1 ROP) [99]. In mouse HAR, activation of the APN pathway is found to be a compensatory response to improve retinal neurovascular development [99]. More interestingly, photoreceptor metabolism is reported to control the formation of deep retinal vascular plexus; improving photoreceptor metabolism leads to neurovascular protection in mouse HAR [99]. Neural control of retinal vascular stability and growth [3,100,101] is based on metabolic demands of neurons dictating growth (or loss) of vessels to supply oxygen and nutrients. Photoreceptors have the highest density of mitochondria and the highest energy demand of any cell in the body [102]. Photoreceptor energy demands are likely a major driving factor for vessel growth [3,99]. Thus, the HAR model makes it possible to explore the risk factors for ROP progression and expand our current understanding of retinal metabolism in neurovascular function.

## 4. Regulation of Retinal Metabolism

### 4.1. Nutrients

#### 4.1.1. Glucose

Glucose metabolism is one of the most important factors controlling endothelial cell (EC) proliferation, migration, and neovascularization [103,104,105]. Blood-derived glucose penetrates the RPE and the blood–retinal barrier and arrives at the retina facilitated by sodium-independent glucose transporter 1 (Glut1) generating ATP by aerobic glycolysis [106]. ECs rely on glycolysis rather than OXPHOS for ATP production and vessel sprouting, and ECs nearly double their glycolytic flux, particularly in tip cells exposed to angiogenic stimuli, such as VEGF [107]. Glycolysis in ECs is modulated by the rate-limiting enzyme, 6-phosphofructo-2-kinase/fructose-2,6-biophosphatase 3 (PFKFB3). Pharmacological inhibition of PFKFB3 or EC-specific genetic deletion of *Pfkfb3* inhibits pathological retinal neovascularization in mouse OIR [108,109]. Promotion of glucose uptake during hyperoxia in rat OIR through the inhibition of mitochondrial uncoupling protein 2 (UCP2), a cellular glucose regulator that decreases glucose uptake through Glut1, attenuates the retinal vaso-obliteration and subsequent neovascularization [110]. The adenosine A2a receptor (ADORA2A) promotes HIF-1-dependent endothelial cell glycolysis, and the EC-specific *Adora2a* deletion decreases retinal neovascularization in mouse OIR [111]. In addition, under physiological conditions, glycolysis converts glucose to energy, with less than 3% of glucose diverted into the polyol pathway, which reduces glucose to sorbitol and increases oxidative stress through the production of highly toxic advanced glycation end products [112]. Aldose reductase is the rate-limiting enzyme in the polyol pathway, and the deletion of the enzyme reduces retinal neovascularization through the attenuation of oxidative stress and protects retinal neurons in mouse OIR [113,114]. These findings suggest that targeting retinal glucose metabolism is an effective way to control pathological retinal angiogenesis.

Recently, single-cell RNA sequencing reveals that glycolysis gene expression is upregulated in proliferating ECs, but less in tip and immature ECs in a mouse model of choroidal neovascularization [115]. Proliferating ECs also upregulated genes involved in one-carbon metabolism, nucleotide synthesis, TCA cycle and OXPHOS [115], suggesting the involvement of other metabolic pathways in modulating pathological ocular angiogenesis. Further exploration of their role in ROP is needed.

#### 4.1.2. Amino Acids

Premature infants frequently lack arginine and glutamine because they are unable to maintain the endogenous synthesis of these conditionally essential amino acids [116,117]. The supplementation of arginine and glutamine (Arg-Gln) suppresses pathological neovascularization in OIR; an in vitro experiment in human RPE cells showed that Arg-Gln decreases VEGF expression [63]. ECs have high glutaminase (GLS) activity, which is the enzyme that converts glutamine and glutaminase in the first and rate-limiting step of glutaminolysis, producing energy for proliferation [118]. Glutamine is indispensable for vessel sprouting, and the inhibition of GLS1 causes sprouting defects in vitro and in mouse models of developmental angiogenesis and pathological neovascularization in OIR in vivo [119].

Serine metabolism via phosphoglycerate dehydrogenase (PHGDH), a key enzyme in the serine synthesis pathway, is important for retinal cell survival, including in EC [80,120]. Loss of *Phgdh* in ECs cause defects in retinal angiogenesis and promotes EC apoptosis via heme deficiency, which induces mitochondrial respiration defects and oxidative stress [121]. Activation of serine and one carbon metabolism is required for HIF-1 stabilization to protect against hyperoxia-induced retinal vaso-obliteration in mouse OIR [78]. Meanwhile, disruption of serine synthesis in the Müller glia also induces mitochondrial dysfunction [122] and the Müller glia relies on serine biosynthesis to combat oxidative stress [123]. Müller glia is the primary source of VEGF in neovascular retina [124,125]. Therefore, targeting retinal serine metabolism may protect against retinal neovascularization in ROP.

#### 4.1.3. Fatty Acids

Fatty acids are the other major substrate for energy production in ECs. In vitro, glucose deprivation causes ECs to increase fatty acid oxidation (FAO) flux in an AMP-activated protein kinase (AMPK)-dependent manner [103]. Endothelial FAO plays an important role in regulating vessel sprouting [126]. As the rate-limiting enzyme of FAO, carnitine palmitoyltransferase 1a (CPT1a) imports FAs into the mitochondria. The endothelial loss of CPT1a causes retinal vascular sprouting defects due to impaired proliferation (not migration) through the inhibition of de novo nucleotide synthesis for DNA replication [126]. ECs express fatty acid synthase (FAS), and FAS-mediated de novo lipogenesis is required for vascular sprouting and permeability [127]. VEGF enhances the expression of fatty acid uptake and trafficking protein FABP4, which is required for normal EC proliferation [128]. Moreover, decreases in both FAO and glycolysis in photoreceptors also induces HIF stabilization and VEGF production, resulting in retinal neovascularization in mice [3,129]. These findings suggest modulating retinal FAO may also prevent neovascular ROP.

### 4.2. Hormones

#### 4.2.1. Adiponectin (APN)

APN is an abundant circulating adipokine involved in metabolic modulation [2]. In premature infants, low circulating APN levels correlate with delayed retinal vascularization and ROP progression [9]. In mouse OIR, loss of APN exacerbates and APN administration decreases retinal neovascularization [130]. Loss of APN receptor 1 in mice leads to abolished DHA uptake, retention, conservation, elongation in photoreceptors, and eventual photoreceptor degeneration [131,132]. In mouse HAR, pharmacologic activation of the APN pathway by recombinant APN or APN receptor agonist exerts protective effects on retinal vessel growth and neuronal development [99]. These studies suggest that increasing circulating APN levels might benefit the preterm infants and decrease the risk for ROP incidence and progression.

Omega-3 LCPUFA increases circulating APN, which mediates omega-3 LCPUFA’s inhibitory effects on neovascularization in OIR mice [9], as well as in other mouse models with proliferative retinopathy [133]. In premature infants, circulating APN is positively correlated with DHA [9]. The increase in circulating APN by dietary omega-3 LCPUFA has also been demonstrated in various studies [134,135,136,137]. These reports suggest that omega-3 LCPUFA supplementation is essential in maintaining circulating APN levels to prevent ROP.

In addition, APN levels could be modulated by fibroblast growth factor 21 (FGF21) [138], which is expressed in many tissues but mainly in the liver under physiologic conditions [139]. FGF21 plays an essential role in modulating lipid and glucose use [140,141,142]. FGF21 is also a key regulator of browning of white adipose tissue and increases energy expenditure [143]. FGF21 via APN inhibits choroidal and retinal neovascularization in mice [144]. FGF21 also increases APN secretion in obese mice [138] and protects diabetes-induced retinal neuronal dysfunction [145]. Furthermore, FGF21 preserves retinal neuronal responses in mice with inherited retinal degeneration [146]. In preterm infants, circulating FGF21 levels are very low, and the postnatal increase in FGF21 observed in full-term infants seems absent in preterm infants [147,148,149]. Taken together, these reports suggest that circulating FGF21 levels may be correlated with increase in APN levels and ROP progression in preterm infants. Further clinic investigations are needed to validate this hypothesis.

#### 4.2.2. Insulin-Growth Factor 1 (IGF-1)

IGF-1 is an important liver-derived growth factor and a key regulator of body growth and development [150,151]. In premature infants, persistent low circulating IGF-1 levels strongly correlate with ROP development [94,152,153,154,155,156]. IGF-1 is critical for normal retinal vascularization as a lack of IGF-1 in mice prevents retinal vessel growth [152]. IGF-1 also supports VEGF activation of endothelial cell proliferation [152,157]. Therefore, early restoration of IGF-1 may prevent ROP. Mice with early supplementation of IGF-1 before exposure to hyperoxia have less vessel loss and neovascularization in the OIR model [158]. In premature infants with postnatal hyperglycemia in the first month, there are also lower plasma IGF-1 levels [94]. In mouse OIR model combined with the HAR model, decreased liver IGF-1 expression is observed before the induction of hyperglycemia; IGF-1 treatment reduces retinal neovascularization and improves retinal revascularization [94]. These findings suggest that early supplementation of IGF-1 may improve retinal vascularization and decrease ROP risk. The phase 2 randomized controlled trial (ClinicalTrials.gov Identifier: NCT01096784) shows that rhIGF-1/rhIGFBP-3 decreases the occurrence of severe bronchopulmonary dysplasia, but the dose needs to be further optimized for ROP prevention [159]. Increasing the number of patients in the study would also help evaluate the effects of IGF-1 on ROP with completion of the current phase 2b clinical trial using SHP607 (recombinant protein complex of IGF-1/IGFBP3) in preterm infants (ClinicalTrials.gov Identifier: NCT03253263). Moreover, recent investigations have also demonstrated that low circulating IGF-1 levels are correlated with low weekly platelet counts [156], which is associated with ROP progression in premature infants [160,161]. Platelet transfusions inhibit retinal neovascularization in OIR mice [160], suggesting that normalizing platelet levels and platelet-derived growth factors (IGF-1, VEGFA, PDGFBB [156]) might prevent ROP in premature infants.

### 4.3. Other Related to Metabolism

#### 4.3.1. Peroxisome Proliferator-Activated Receptor α (PPARα) Agonist

Fenofibrate, a PPARα agonist, is an antihyperlipidemic drug. The FIELD and ACCORD studies have shown that fenofibrate suppresses the progression of diabetic retinopathy [162,163]. Fenofibrate modulates lipid metabolism, reduces triglyceride (TG), and increases high-density lipoprotein (HDL) cholesterol [164]. Fenofibrate inhibits neovascularization in OIR mice through the suppression of HIF-1α and VEGF [165]. However, fenofibrate is not recommended for patients with renal dysfunction because it metabolizes in the kidney. However, kidneys are often underdeveloped in premature infants [166]. Recently, pemafibrate, which is a selective PPARα modulator, has been approved for use in Japan. Pemafibrate is as effective as fenofibrate in modulating hyperlipidemia and reduces the associated risks in the liver and kidney [167,168], possibly due to the structural differences between fenofibrate and pemafibrate [169]. Pemafibrate decreases retinal neovascularization in OIR mice and protects retinal function in diabetic mice model by inducing FGF21 [170,171]. Pemafibrate also suppresses HIF1α and *Vegf* in OIR retinas [170]. Currently, phase 3 clinical trials of the use of pemafibrate to reduce cardiovascular outcomes by reducing triglycerides in patients with type 2 diabetes (PROMINENT) is ongoing (ClinicalTrials.gov Identifier: NCT03071692). With the potential application of pamafibrate in treating diabetes and diabetic retinopathy, pamafibrate may also be a therapeutic potential for other retinal metabolic disorders such as ROP.

#### 4.3.2. Rapamycin

Rapamycin (Sirolimus) is an inhibitor of mammalian target of rapamycin (mTOR), with anti-proliferative, antiangiogenic, and immunosuppressive properties [172]. Rapamycin is used to prevent organ transplant rejection and treat lymphangioleiomyomatosis, a rare lung disease [173,174]. mTOR is a serine-threonine protein kinase and functions as two distinct signaling complexes: mTOR complex 1 (mTORC1) and mTORC2 [175]. mTORC1 is involved in immune responses and lipid metabolism in the human body [176]. In the context of eye disease, several studies showed that systemic rapamycin treatment reduces retinal neovascularization in OIR mice [177,178]. Rapamycin also reduces vascular apoptosis and promotes proliferation and tip cell function in OIR mice [179]. Together, these data suggest that rapamycin may be a promising strategy for early intervention of ROP.

#### 4.3.3. Rho-Associated Kinase (ROCK) Inhibitor

ROCK is involved in inflammation, angiogenesis, apoptosis, and cytoskeletal rearrangement [180,181,182]. ROCK is identified as a downstream effector of the small GTP-binding protein Rho and has two isoforms, ROCK1 and ROCK2 [183]. Noda et al. showed that inhibition of Rho-kinase increases energy expenditure via AMPK activation in brown adipose tissue and improves metabolic disorders [184]. Several ROCK inhibitors exhibit suppression of pathological neovascularization in OIR rodent models up to date, such as Fasudil, Ripasudil, Y27632, and AMA 0428 [185,186,187]. Ripasudil, in particular, induces pericyte coverage and improves retinal vascular perfusion in mouse OIR [186]. Several clinical trials are currently active to evaluate whether Fasudil or Ripasudil eye drop affects ROP prevention (ClinicalTrials.gov Identifier: NCT04191954, NCT04621136). Topically applied ROCK inhibitors would be potentially beneficial for ROP treatment.

#### 4.3.4. Autophagy

Autophagy is a cellular process induced by many stresses, including hypoxia, starvation, and infection, to maintain homeostasis [188]. Autophagy increases during the first postnatal days and decreases as the retina reaches full vascularization in rats [189]. Elevated retinal reactive oxygen species and attenuated autophagy is shown in OIR mice [190]. Knockdown of β5i, an immunosubunit of the immunoproteasome, increases autophagy-related protein 5 (ATG5) and inhibits retinal neovascularization in mouse OIR [191]. On the other hand, a significant increase in autophagy flux is reported in mouse OIR retinas, particularly in proliferating endothelial cells [192]. Endothelial-specific deletion of ATG5 attenuates retinal neovascularization in OIR mice [193]. Further studies are needed to elucidate the role of autophagic imbalance in ocular angiogenesis. Overall, autophagy could be a novel target for pharmacological intervention in ROP patients.

## 5. Future Perspectives

**Figure 4:**
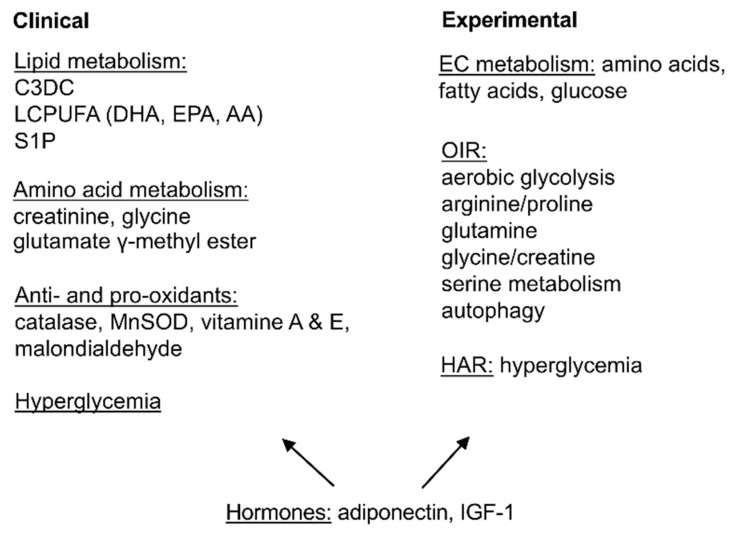
Summarized current findings of metabolism in ROP. Lipid and amino acid metabolic disturbance, hyperglycemia as well as unbalanced antioxidant system were found. Hormones including adiponectin and IGF-1 are essential in modulating metabolic responses. The combination of nutrients and the timing of nutritional and hormonal intervention, as well as the corresponding specific cell responses need to be carefully evaluated.

We here summarized the current understanding of metabolic impacts on ROP (Figure 4). Overall, metabolic disturbances in glucose, amino acid, and lipid use may contribute to ROP development and progression. Hormonal modulation plays an essential role in maintaining metabolic homeostasis. Further understanding of the relationship and interaction among risk factors at early and late stages of ROP is essential for clinical intervention. Our current knowledge gained from DHA and AA supplementation in premature infants suggests that maintaining adequate AA levels is required for DHA to exert protective effects against ROP [25,30,194]. In addition, loss of APN largely abolishes DHA’s role of inhibition on retinal neovascularization in mouse OIR [9]. Restoration of APN levels in premature infants may further exaggerate DHA protection against ROP. Moreover, there is a potential concern that continuous peroxidation of VLCFA may cause chronic inflammation and neuronal damage [195,196]. Thus, the timing in the administration of DHA and AA intervention also needs to be evaluated. The same concept also applies to IGF-1 supplementation (and potentially other therapeutic targets as well) as IGF-1 improves retinal vascularization in phase I ROP and may exacerbate VEGF-induced neovascularization at phase II ROP [152,157].

To expand our current knowledge of nutritional and hormonal regulation in retinal metabolism and ROP, we need to further understand the types of metabolic substrates in retinal neuronal and endothelial cells, as well as the interaction among the different types of retinal cells. Endothelial cell metabolism (glycolysis, fatty acid oxidation, and serine synthesis) controls physiological and pathological retinal angiogenesis [103,121], which may be controlled by Müller glia [125,197]. Müller glial cells also produce and transfer nutrients (such as lactate) to photoreceptors [198] in addition to the uptake and conversion of glutamate to glutamine [199]. Moreover, retinal pigmented epithelium maintains photoreceptor metabolism by transferring glucose and passing and recycling lipids to photoreceptors [200,201,202,203,204,205,206]. Photoreceptor metabolism also controls physiological and pathological retinal vessel growth [3,99]. Taken together, there are complex interactions among retinal cells. Specific cellular responses and cell-cell interaction need to be considered and long-term impacts need to be examined.

## Data Availability

Not applicable.
